# Using geographic methods to inform cancer screening interventions for South Asians in Ontario, Canada

**DOI:** 10.1186/1471-2458-13-395

**Published:** 2013-04-26

**Authors:** Aisha K Lofters, Piotr Gozdyra, Rebecca Lobb

**Affiliations:** 1 Department of Family & Community Medicine, University of Toronto, Toronto, Canada; 2 Department of Family & Community Medicine, St. Michael’s Hospital, Toronto, Canada; 3 Centre for Research on Inner City Health, The Keenan Research Centre in the Li Ka Shing Knowledge Institute of St. Michael’s Hospital, Toronto, Canada; 4 Institute for Clinical Evaluative Sciences, Toronto, ON, Canada; 5 Division of Public Health Sciences, Department of Surgery and Alvin J. Siteman Cancer Center, Washington University School of Medicine, St Louis, MO 63110, USA

## Abstract

**Background:**

Literature suggests that South Asians in Ontario, Canada are under-screened for breast, cervical and colorectal cancer. Accordingly, we are involved in a community-engaged multi-phase study aimed at increasing cancer screening for this vulnerable group. In the work described in this manuscript, we aimed to use visual displays of spatial analyses to identify the most appropriate small geographic areas in which to pilot targeted cancer screening interventions for Ontario’s South Asian community.

**Methods:**

We used Geographic Information Systems (GIS), including Local Indicators of Spatial Association (LISA) using GeoDa software, and population-level administrative data to create multi-layered maps of: i) rates of appropriate cancer screening, ii) the percentage of residents of South Asian ethnicity, and iii) the locations of primary care practices and community health centres by census tract in the Peel Region of Ontario (population: 1.2 million). The maps were shared with partner health service and community service organizations at an intervention development workgroup meeting to examine face validity.

**Results:**

The lowest rates of appropriate cancer screening for census tracts across the region were 51.1% for cervical cancer, 48.5% for breast cancer, and 42.5% for colorectal cancer. We found marked variation both in screening rates and in the proportion of South Asians residents by census tract but lower screening rates in the region were consistently associated with larger South Asian populations. The LISA analysis identified a high-risk area consisting of multiple neighbouring census tracts with relatively low screening rates for all three cancer types and with a relatively large South Asian population. Partner organizations recognized and validated the geographic location highlighted by the LISA analysis. Many primary care practices are located in this high-risk area, with one community health centre located very nearby.

**Conclusions:**

In this populous region of Ontario, South Asians are more likely to reside in areas with lower rates of appropriate breast, cervical and colorectal cancer screening. We have identified a high-risk area appropriate for both patient- and provider-focused interventions. Geographic Information Systems, in particular LISA analyses, can be invaluable when working with health service and community organizations to define areas with the greatest need for interventions to reduce health inequities.

## Background

In Ontario, Canada’s largest province, a growing body of literature suggests that South Asians (i.e. people from India, Pakistan, Bangladesh and Sri Lanka) are vulnerable to under-screening for breast, cervical and colorectal cancer. Both breast and cervical cancer screening rates have been found to be lower among women who live in low-income or high-immigration areas [1-3]. Research has also shown that South Asian women in Ontario have breast and cervical cancer screening rates lower than those of other immigrant groups [4,5]. In Ontario, colorectal cancer screening rates are universally low [6], and low income, non-white ethnicity, and being foreign-born have each been independently associated with lower odds of colorectal cancer screening at the national level [7]. South Asians are one of the fastest growing immigrant groups in Canada and Ontario, and are over-represented in the lowest-income neighbourhoods, suggesting that they are a group particularly vulnerable to being inadequately screened for all three types of cancer [5,8,9].

We are currently involved in a multi-phase community-engaged research collaboration whose ultimate aim is to institute a sustainable and effective breast, cervical and colorectal cancer screening intervention for Ontario’s South Asian community. We are initiating our study in Peel Region. According to the 2006 Census, Peel Region has a population of 1.15 million people, and the largest South Asian population in the province. In the first phase of the study, we used concept mapping to obtain a community-generated list of barriers to cancer screening among South Asians in Peel [10]. In Phase II, we are working with a community advisory group, consisting of primary care providers, community service agency representatives, and representatives of public health and health service organizations who all function in Peel, to select evidence-informed intervention strategies to address these barriers. The community advisory group will work with us to adapt and implement these intervention strategies with selected local community partners, such as health service organizations and primary care providers. Selected strategies will be piloted on a small scale and formatively evaluated to assess reach, acceptability and feasibility.

Toward that end, in the current paper we sought to use GIS to simultaneously map cancer screening rates, the size of the South Asian population, and the locations of primary care practices and community health centres by census tract (CT) in Peel Region. GIS technology allows for the integration of health data with mapping functions and for the visualization of health outcome patterns, and can play a major role in identifying locations where interventions are needed [11-13]. We also sought to use Local Indicators of Spacial Association (LISA) analysis to identify the most high-risk areas in Peel Region. LISA analysis typically allows for identification of hot spots, where screening rates are higher or lower than what would be expected by chance alone [14].

**Figure 1:**
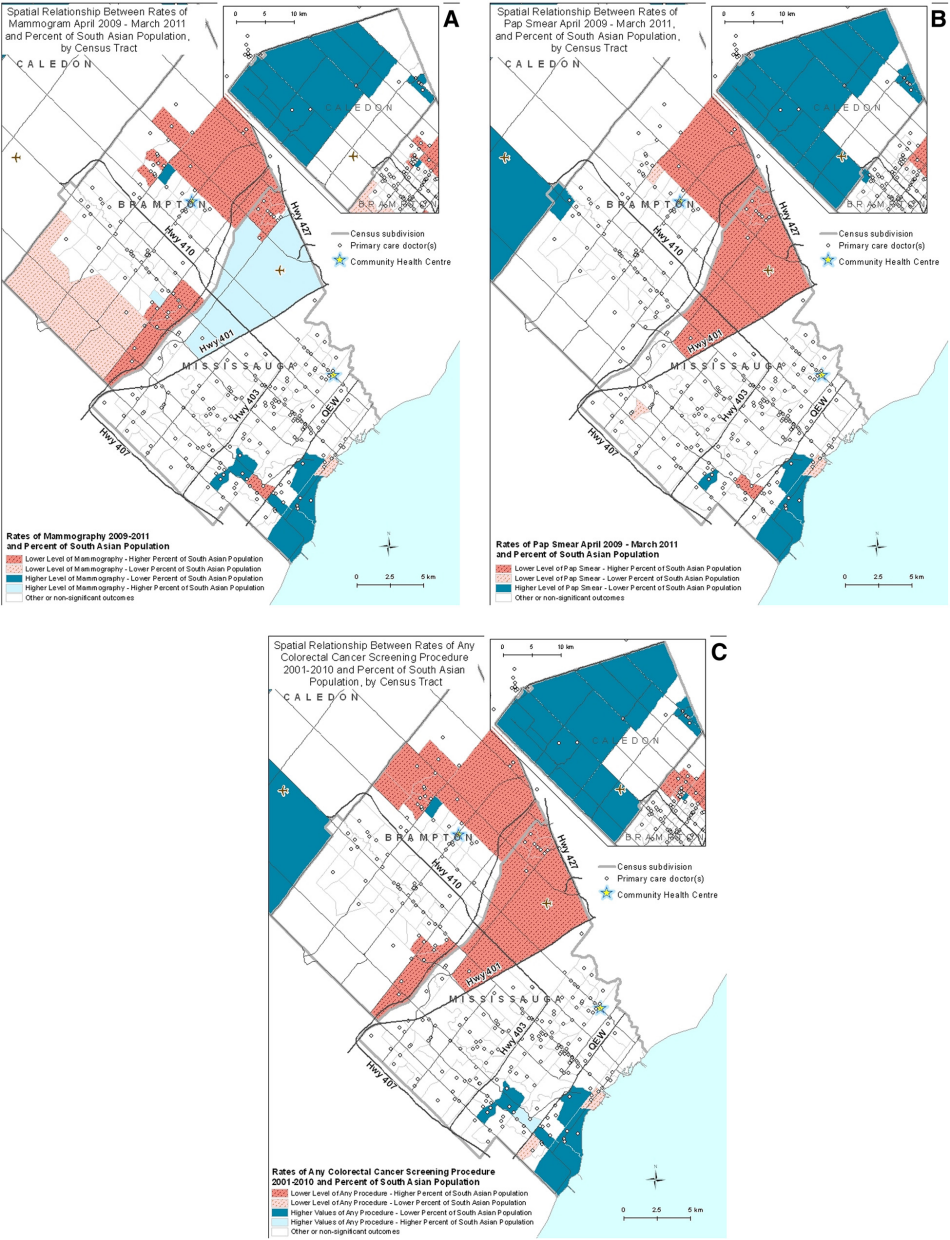
Local indicators of spatial association maps for cancer screening and South Asian ethnicity.

We used univariate LISA analysis for rates for each of the types of cancer screening, and for the percentage of South Asian population. We then overlaid the statistically significant outcomes (p < .05) identifying the areas of most concern where the cancer screening rates were low and the percentage of South Asian population were high. We also identified the areas of least concern where the screening rates were high and the percentage of South Asian population was low, as well as areas where both rates were high or low. The resulting maps (Figure 1A-C) aimed to identify areas within Peel in which to pilot our interventions. The ideal target area would have significantly lower screening rates for breast, cervical and colorectal cancer and would have a significantly higher proportion of its population identifying as South Asian.

## Methods

### Study setting

According to the 2006 Canadian Census, there were 1 159 405 people living in Peel Region. Peel contained a total of 207 CTs, 205 of which had both ethnicity information and cancer screening information available from the census. Peel is divided into three Census subdivisions: Caledon, Brampton and Mississauga.

We accessed information about Peel Region’s population eligible for health services and adults receiving cancer screening through a comprehensive research agreement with Ontario’s Ministry of Health and Long-Term Care. The research protocol was approved by the Research Ethics Board at Sunnybrook Hospital in Toronto.

### Data sources

Several databases were accessed for this study. Ontario’s health care registry, the Registered Persons Database (RPDB), includes all Ontario residents eligible for health coverage by age, sex and address. To be eligible for health coverage, one must be a Canadian citizen, permanent resident, or convention refugee, make one’s permanent and principal home in Ontario, and be physically present in Ontario for 153 days in any 12-month period. The Ontario Health Insurance Plan (OHIP) Physicians’ Claims Database contains claims for physician and hospital services and includes approximately 95% of physician claims in the province [15]. The Ontario Cancer Registry is a registry of all Ontario residents who have been newly diagnosed with cancer or who have died of cancer. The Canadian Institute of Health Information Discharge Abstract Database contains fee codes and corresponding diagnostic codes claimed by Ontario’s physicians. Cytobase is Ontario’s electronic Pap test registry. The Ontario Breast Screening Program (OBSP) data record date of mammography for all women who participate in the province’s breast cancer prevention program. The Corporate Provider Database (CPDB) provides postal codes of primary care providers in the province.

### Determination of rates of appropriate cancer screening by census tract

To determine cancer screening rates by CT, we used the RPDB to identify Peel residents eligible for breast, cervical and colorectal cancer screening based on provincial guidelines. Provincial guidelines by Cancer Care Ontario recommend that women aged 21–70 years be screened at least once every three years for cervical cancer, that women aged 50–69 years be screened at least once every two years for breast cancer, and that adults aged 50 years and over be screened at least once every two years for colorectal cancer.

Using postal codes from the RPDB, residents were assigned to specific 2006 CTs using Statistics Canada’s Postal Code Conversion File Plus [16]. Anyone in the RPDB who had no contact at all with the health care system, including a physician office visit, hospitalization, emergency room visit, or drug benefit claim between April 1, 2008-March 31, 2011 was excluded, as these people are more likely to have died or moved out of the province. Across the screening cohorts (described below), an average of 6.5% of people were excluded due to lack of contact in the RPDB.

Eligibility for colorectal cancer screening was defined as being alive, living in Ontario, and 52–74 years of age on April 1, 2011. Anyone who had ever been diagnosed with colorectal cancer or severe inflammatory bowel disease was excluded. A total of 276 314 Peel residents were eligible. We used three definitions of appropriate colorectal cancer screening. People were considered appropriately screened if they had: i) FOBT between April 1, 2009 and March 31, 2011, or ii) colonoscopy between April 1, 2001 and March 31, 2011, or iii) sigmoidoscopy or barium enema between April 1, 2006 and March 31, 2011.

Eligibility for breast cancer screening was defined as being female, alive, living in Ontario and 52–69 years of age on April 1, 2011. Anyone who had ever been diagnosed with breast cancer was excluded. A total of 120 111 women were in this cohort. Women were considered appropriately screened if they had a mammogram between April 1, 2009 and March 31, 2011.

Eligibility for cervical cancer screening was defined as being female, alive, living in Ontario and 24–69 years of age on April 1, 2011. Anyone who had ever been diagnosed with cervical cancer, or who had any record of a hysterectomy or colposcopy, was excluded. There were 333 072 women in this cohort. Women were considered appropriately screened if they had a Pap test between April 1, 2008 and March 31, 2011.

### Determination of ethnicity by census tract

2006 Census data were used to determine the ethnic origins of residents of each CT, namely, the proportion of residents of each CT who were identified as being of South Asian ethnicity. The South Asian region includes India, Pakistan, Bangladesh, and Sri Lanka.

### Creation of maps

Two versions of each map were created. Choropleth (shaded) maps were used to depict the proportion of eligible adults in each CT who had each form of cancer screening (cervical, breast, colorectal), where the intensity of shading indicated the magnitude of screening. Circles were then overlaid, proportionate in size to the proportion of the population who identified as being of South Asian ethnicity.

Maps depicting results of Local Indicator of Spatial Association (LISA) analyses were also created. The LISA analysis used local Moran’s I indicator at the 0.05 significance level. These maps showed overlaid outcomes of two univariate LISA analyses, which identified clusters of high (or low) cancer screening rates and high (or low) percentages of South Asian populations.

In all maps, dots and stars, respectively representing locations of family physician practices and community health centres, were overlaid based on postal codes from the CPDB.

## Results

Demographic characteristics of the study area from the 2006 Census are summarized in Table 1 and compared to the province as a whole. Peel Region had a noticeably higher proportion of its population who were foreign-born. Approximately four times the proportion of residents of Peel as compared to residents of Ontario spoke a South Asian mother tongue, were born in South Asia, were recent South Asian immigrants, and were of South Asian ethnicity. Median household income was higher in Peel Region than the rest of the province, but the proportion of population with no certificate, diploma and degree was similar.

**Table 1:** Demographic profile of Peel Region and the province of Ontario, based on 2006 Census data

	**Peel Region**	**Ontario**
Population, n	1,159,405	12,160,282
First language South Asian*,%	16.7	4.4
Knowledge of English,%	96.2	97.4
Foreign-born,%	48.6	28.3
Born in South Asia,%	14.6	3.9
Born in South Asia and immigrated in preceding 5 years,%	5.3	1.3
South Asian ethnicity,%	23.6	6.6
Median household income, Can$	72,655	60,455
Population 25–64 years with no certificate, diploma or degree,%	12.3	13.6

*Bengali, Gujarati, Hindi, Punjabi, Sindhi, Sinhalese, Urdu, Malayalam, Tamil, Telugu.

In Table 2, screening rates for Peel Region overall are compared with those for Ontario as a whole. For all cancer types and screening modalities, rates in Peel were slightly lower than those for the province. The pattern of lowest to highest screening for specific tests was similar for Peel Region and Ontario.

**Table 2:** **Percentage of screen-eligible population who were screened within appropriate time frame**^**+ **^**for Peel Region and Ontario**

	**Peel Region**	**Ontario**
Cervical cancer screening	66.3	67.6
Breast cancer screening	61.3	63.4
Fecal occult blood testing	29.5	32.1
Colonoscopy	35.4	37.0
Any colorectal cancer screening*	55.6	58.3

^+^Defined as three years for cervical cancer screening, two years for breast cancer screening, two years for fecal occult blood testing, ten years for colonoscopy, five years for barium enema, five years for sigmoidoscopy. End date for all time frames was April 30, 2011.

*Any colorectal cancer screening = fecal occult blood testing, colonoscopy, barium enema, sigmoidoscopy.

**Figure 2:**
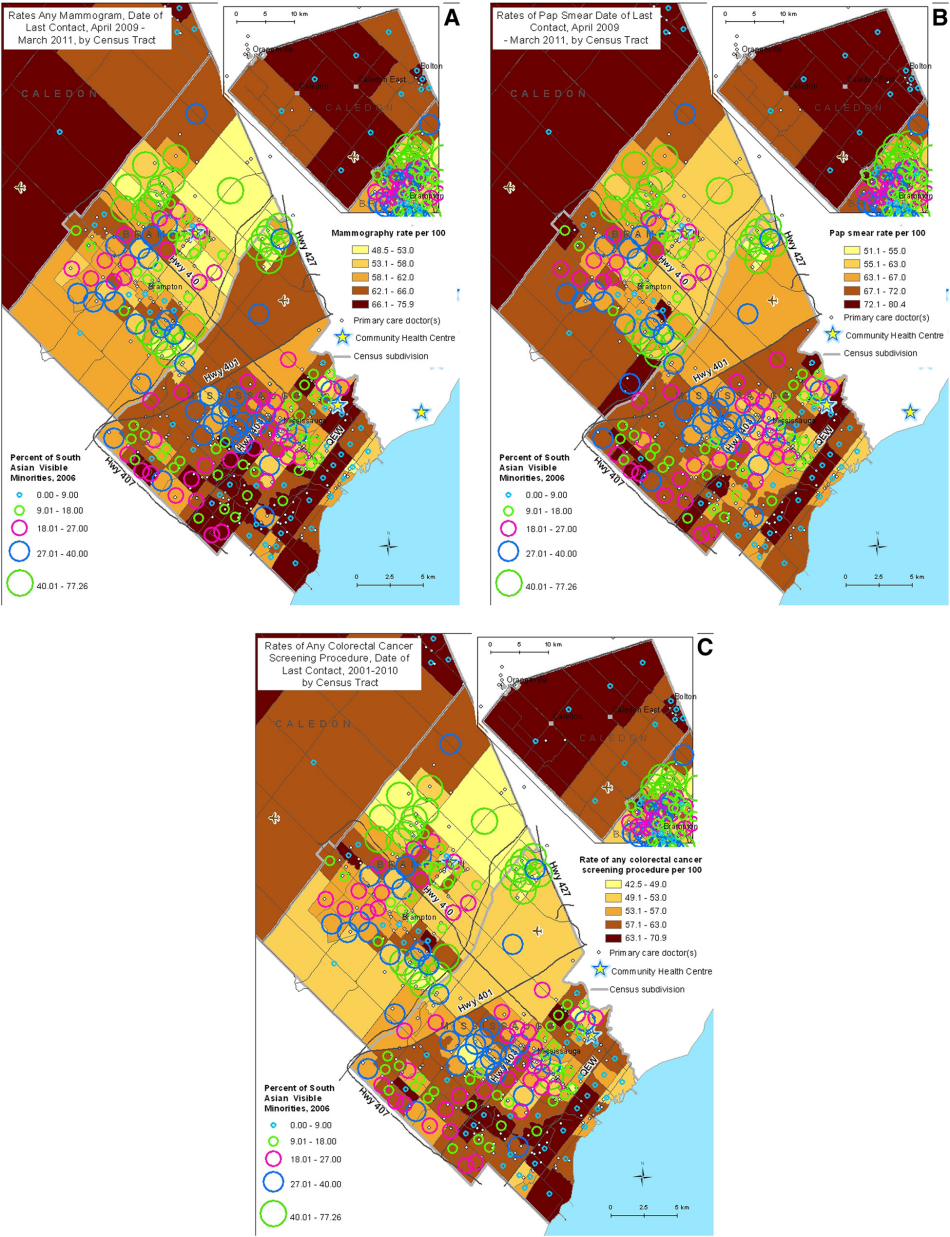
Cancer screening rates overlaid with South Asian population.

Our maps of Peel demonstrate marked variation in cancer screening rates by CT. Spatial patterns (Figure 2A-C) demonstrate that, across Peel Region, lower screening rates were consistently associated with larger South Asian populations for all three cancer types. Screening rates for CTs in Peel were as low as 51.1% for appropriate cervical cancer screening, 48.5% for appropriate breast cancer screening, and 42.5% for appropriate use of any colorectal cancer screening procedure. A particularly vulnerable area seemed apparent in eastern Brampton and northeastern Mississauga. The multiple CTs included in this area consistently had among the lowest screening rates and had at least 40% of their population self-identifying as South Asian. Several primary care practices are located in this area, and one of the two community health centres in Peel Region is just west of this vulnerable area.

The LISA maps (Figure 1A-C) provided a clearer and more scientifically rigorous definition of the same vulnerable area of the region. On all LISA maps, eastern Brampton and the northeastern part of Mississauga simultaneously showed significantly lower rates of cancer screening and higher rates of South Asian populations. Areas with the opposite outcome, higher rates of cancer screening and lower rates of South Asian populations, were fairly consistently present within Caledon and across southern and western parts of Mississauga. Areas with lower rates of both characteristics and areas with higher rates of both characteristics were rare. The exception to this was an area in northeast Mississauga where there was a higher level of mammography use and a larger South Asian population.

## Discussion

Using GIS, we have identified a particular area in Peel Region which has a large South Asian population as well as consistently low screening rates for each of breast, cervical, and colorectal cancer. From the LISA analysis, we know that these spatial differences are statistically significant and are not accounted for by chance alone. By visually depicting both cancer screening rates and the size of the South Asian population by CT, the maps that we created have highlighted this and other areas in Peel Region in particular need of intervention. These maps also illustrate physician practices and community health centres that are located in or near these most vulnerable areas. The presence of these practices suggests that it would be reasonable to develop multi-level interventions, targeted at both patients and physicians, and that these primary care practices should be considered as crucial implementation partners as we move forward with the next phases of our study. Community service organizations and health service organizations that are located in these most vulnerable areas will also be important potential implementation partners for targeted interventions for South Asian residents of the region. In the future, these maps can be re-generated as one method to monitor the effectiveness of our implemented interventions in particular CTs.

Our community advisory group confirmed the face validity of the results of our maps, with some member organizations being situated within or quite near to the borders of the identified high-risk area. For those members who did not function in the highlighted area, the compelling nature of the results precluded any disagreement on the best location within Peel Region to pilot selected interventions. Despite being based on a complicated geographic and analytical concept, all advisory group members were able to easily understand the implications of the LISA maps because of the visual representation.

The use of GIS to visually depict cancer-relevant health data is well documented in the cancer literature [17-23], and geographic methods have been used to examine the association between cancer screening and sociodemographic characteristics in several studies [2,3,11,24]. However, we found no other studies that used GIS across the three evidence-based types of cancer screening for one vulnerable population, or that used LISA to clearly delineate the most high-risk areas for cancer screening for a vulnerable population. Similar to our community-based approach, Cromley et al. used GIS and worked with community members to identify a small target geographic area for interventions to reduce health inequities, in their case focussed on diabetes [25]. The authors emphasized that available resources could have maximum impact and that knowledgable local organizations could collaborate substantively by focussing initially on a well-defined small geographic area. Gwede et al. also used GIS in the context of a community-academic collaborative to map colorectal cancer screening resources in relation to the local African-American population [11]. They were thus able to identify where additional colonoscopy resources were needed.

Our results strongly suggest that South Asians in this region of Ontario are under-screened for all three forms of cancer. This is in line with other Canadian and Ontario literature which have demonstrated low breast and cervical cancer screening rates for women of South Asian ethnicity and South Asian immigrants [4,5,26-29]. We found no studies that investigated colorectal cancer screening for South Asians in Canada. Not surprisingly, South Asian immigrants in the UK and the US have also been found to have lower screening rates for all three cancers than would be expected by guidelines, and lower than those of their non-South Asian peers [30-34]. Our results also highlight an area in Peel Region where appropriate breast cancer screening and a South Asian population are both prevalent. As we move forward, the reasons for this small but meaningful success story, and why it only applies to one cancer type, will have to be explored with South Asian residents, primary care providers and organizations within this area.

Although our method of combining population-level administrative data with spatial analysis provides us with important information on areas in Peel Region that may be most appropriate to initiate cancer screening interventions for the South Asian community, it has several limitations. First, our findings are subject to ecological fallacy because the data were measured at the area level and not at the patient level. If a particular CT has low cancer screening rates and a high South Asian population, that is not direct evidence that the South Asians living in that CT have low cancer screening rates. Similarly, we must be cognizant that primary care practices in the highlighted area may not serve South Asian residents or have low rates of cancer screening, that the catchment area of some practices might not be the same as the area immediately surrounding the practice, and that they may draw their patients from the local population in an unrepresentative way. Second, reporting a change of address to Ontario’s Ministry of Health and Long-Term Care is voluntary, and deaths are not immediately updated in the databases we used. Therefore, we may have incorrectly assigned people to particular CTs or incorrectly assumed them to be alive. However, we limited our population to those who had a date of contact with the health care system from April 1, 2008 to March 31, 2011. It must be noted that this does not guarantee that the individual still lives within the particular CT to which they have been assigned. Third, we chose to use CTs, as opposed to smaller dissemination areas, as our unit of analysis. The CTs are of varying sizes, and some can be quite large, accordingly with heterogeneity within their borders. However, dissemination areas can be as small as the size of an apartment building, and therefore would have been difficult to map, may have led to unstable rates, would have been too small to provide meaningful conclusions about physician practices to target, may frequently have had missing information about screening rates and the South Asian population, and may have been too small to accurately monitor effectiveness. The CTs in Ontario have an average size of 4000 people (range 2500 to 8000) [35]. Fourth, data on South Asian ethnicity were only available from the 2006 Census. It is possible that the proportion of South Asians living within a CT might have changed since that time. However, considering our compelling findings, and face validation by our community advisory group, we believe that significant differences are unlikely. Finally, the marked variation that we observed in cancer screening rates across CTs may partially reflect small denominators in some CTs.

These data will not be the only determinants of where and with whom our cancer screening interventions are piloted. We are also conducting an organizational network analysis to document and examine the current relationships between local organizations that provide health promotion or community building programs for Peel Region’s South Asian community. Using these results, we will take advantage of opportunities to build on and develop current relationships. However, the maps presented here will play a crucial role in informing where we initiate our interventions in the ongoing study. Our method of spatially analyzing cancer screening rates, ethnicity, and locations of relevant primary care practices and health centres that could be target sites for interventions could be equally beneficial for community-engaged research studies in other settings with access to similar data.

## Conclusions

In the Peel Region of Ontario, we found that South Asians are more likely to live in areas with lower rates of appropriate breast, cervical and colorectal cancer screening. Using Geographic Information Systems, we have identified a high-risk area appropriate for initiating both patient- and provider-focused interventions. Geographic Information Systems, in particular LISA analyses, can be invaluable when working with health service and community organizations to define areas with the greatest need for interventions to reduce health inequities.

## Competing interests

The authors declare they have no competing interests.

## Authors’ contributions

AL took primary responsibility for the design of the study and drafting and revising the article. PG performed all geographic and spatial analyses, and provided advice and direction for the study design. RL took primary responsibility for the conception of the study. She provided advice and direction for the study design. All authors revised the article critically for important intellectual content and gave final approval of the version to be published.

## Pre-publication history

The pre-publication history for this paper can be accessed here:

http://www.biomedcentral.com/1471-2458/13/395/prepub
